# Transcriptomic profile of semitendinosus muscle of bulls of different breed and performance

**DOI:** 10.1007/s13353-020-00577-1

**Published:** 2020-08-26

**Authors:** Anna Ciecierska, Tomasz Motyl, Tomasz Sadkowski

**Affiliations:** 1 Department of Human Nutrition, Institute of Human Nutrition Sciences, Warsaw University of Life Sciences–SGGW, Nowoursynowska 159C, 02-776 Warsaw, Poland; 2 Department of Physiological Sciences, Institute of Veterinary Medicine, Warsaw University of Life Sciences–SGGW, Nowoursynowska 159, 02-776 Warsaw, Poland

**Keywords:** Myogenesis, Skeletal muscle, Protein metabolism, Cattle, Gene expression, Microarray

## Abstract

The aim of the study was to compare the transcriptomic profiles of fully differentiated skeletal muscle derived from bulls belonging to different breeds of varying performance. Microarray analyses were performed to determine the differences in the expression profiles of genes between semitendinosus muscles of 15-month-old beef-breed bulls (Limousin—LIM and Hereford—HER) and dairy-breed bulls (Holstein Friesian—HF). These analyses allowed for the identification of those genes the expression of which is similar and characteristic of fully differentiated muscle in beef breeds, but differs in skeletal muscle of a typical dairy breed. The analysis revealed 463 transcripts showing similar expression in the semitendinosus muscle of beef breeds (LIM/HER), in comparison with the dairy breed (HF). Among the identified genes, 227 were upregulated and 236 were downregulated in beef breeds. The ontological analyses revealed that the largest group of genes similarly expressed in LIM and HER was involved in the processes of protein metabolism and development of muscle organ. In beef breeds, some genes involved in protein synthesis and proteolysis showed an upregulation, including *ctsd*, *ctsf*, *fhl2*, *fhl3*, *fst*, *sirt1*, and *trim63*, whereas some were downregulated, including *bmpr1a*, *bmpr2*, *mstn*, *smad2*, *hspa8*, *gsk3β*, and *tgfβ2*. The expression of the chosen genes was confirmed by RT-qPCR technique. Thus, it can be assumed that the identified genes involved in the regulation of growth and development of muscle tissue and the processes of protein metabolism in the examined cattle breeds may be responsible for the greater gain of muscle mass in beef-breed bulls.

**Electronic supplementary material:**

The online version of this article (10.1007/s13353-020-00577-1) contains supplementary material, which is available to authorized users.

## Introduction

The quality of livestock meat is very important from an economic point of view, and hence, research has been carried out over the past few decades to increase the production of beef, accelerate the growth rate of cattle, and improve the quality of meat (Hocquette et al. 2007; Pires et al. 2017).

Cattle breeds showing different levels of performance are characterized by differences in the structure and physiology of skeletal muscles. The late-maturing Limousin (LIM) cattle breed (with a large proportion of muscle in the carcass and a small amount of adipose tissue) and the early-maturing Hereford (HER) breed (with a large proportion of well-marbled muscle) are both beef breeds, whereas the early-maturing and poorly muscled Holstein Friesian (HF) is a typical dairy breed. The gains of muscle mass up to the 16th month of life are about 1000–1300, 850–1400, and 800–1000 g per day in LIM, HER, and HF breeds, respectively. The reason for these differences observed in the gain of muscle mass is not sufficiently explained, and the genes determining the rate of growth and metabolism of muscle tissue in these breeds remain to be identified (Motyl et al. 2009).

In the last decades, many papers have been published describing the studies using microarray for the investigation of myogenesis-related processes, which enabled the identification of genes affecting the quantity and quality of meat derived from livestock, including cattle (Bernard et al. 2007; Hocquette et al. 2007; Zhao et al. 2012). Sadkowski et al. (2006) studied the differences in gene expression in the semitendinosus muscle of bulls depending on their age. In addition, the authors also performed high-throughput gene expression analysis in the muscle tissue obtained from 12-month-old beef- and dairy-breed bulls (Sadkowski et al. 2009b). Yu et al. (2007) identified the genes showing varied expression in the skeletal muscles of different bovine breeds. Additionally, Lehnert et al. (2007) studied the development of the longissimus muscle in beef cattle breeds, such as Piedmontese, Wagyu, and Hereford. Wang et al. (2005) performed transcriptomic analysis of skeletal muscles obtained from bulls of Japanese Black and Holstein breeds. However, although these microarray studies provided a lot of important information, they generated many questions about the growth and metabolism of skeletal muscles.

The aim of this study was to examine and compare the transcriptomic profiles typical of fully differentiated/matured (15-month-old) skeletal muscle in cattle depending on their breed and performance. The analysis allowed for a better understanding of the genetic regulation of the development of muscle tissue, as well as determining the putative genes involved in myogenesis and development of muscle tissue in beef-breed bulls.

It was assumed that the results obtained will enable the selection of those genes that are involved in the process of myogenesis and maturation of skeletal muscle and modification of their expression for achieving greater gains of muscle mass in beef-breed bulls.

## Materials and methods

### Animals

All the procedures related to animal experiments were approved by the Local Ethics Committee.

The experimental group consisted of bulls belonging to three cattle breeds differing in meat production and performance: LIM (beef breed, capable of high-meat low-fat production; *n* = 4), HER (beef breed, capable of high-meat high-fat production; *n* = 4), and HF (dairy breed; *n* = 4) used as a reference. The bulls were housed, fed, and slaughtered at the age of 15 months as described earlier (Sadkowski et al. 2009a, 2018). The muscle tissue samples were compared individually—4 biological repetitions.

### Tissue sampling, RNA isolation and validation

Tissue sampling (*m. semitendinosus*) and total RNA isolation and validation were done as described in previous papers (Sadkowski et al. 2008, 2009a); briefly, the muscle samples were taken immediately after slaughter, from the central portion of the muscle, dissected free of connective and adipose tissue, and frozen in liquid nitrogen until analyzed. The quality of RNA samples was assessed using Bioanalyzer 2100 (Agilent Technologies, USA). Samples with RIN (RNA integrity number) ˃ 9 were subjected to further analysis.

### Microarray analysis

The gene expression profile was analyzed using Bovine (V2) Gene Expression Microarray 4 × 44 K oligonucleotide slides (Agilent Technologies, USA) and Two-Color Microarray-Based Gene Expression Analysis kit (Agilent Technologies, USA). Probe labeling, hybridization, signal detection, and data extraction were done as described in a previous paper (Wicik et al. 2011). The comparison of transcriptomic profiles of semitendinosus muscle (*m. semitendinosus*) of 15-month-old bulls from beef breeds Limousin (LIM) and Hereford with bulls of the Holstein-Friesian (HF), a dairy breed (reference), was done using the GeneSpring software (Agilent Technologies, USA), *t* test with *p* ≤ 0.05 and fold change ≥ 1.3 as the criteria of significance. Each comparison was performed individually, in four biological repetitions. In the subsequent stage, a comparative analysis of both transcriptomes was performed, aiming at identifying common genes with different expression in the muscles of bulls from beef breeds (LIM, HER). The data obtained from the microarray experiment were deposited in the NCBI (National Center for Biotechnology Information) Gene Expression Omnibus database and numbered GSE137565 (HER vs. HF) and GSE137566 (LIM vs. HF).

### Real-time qPCR

To verify the microarray results, real-time quantitative polymerase chain reaction (qPCR) was applied. The mRNA sequences of the genes selected for validation were obtained from the NCBI Nucleotide database. Primer sequences were designed using the Primer3 and Primer-Blast software (NCBI database) and then checked using Oligo Calculator as described previously (Szcześniak et al. 2016). The sequences of the designed primers are listed in Supplementary Table 1. Glyceraldehyde-3-phosphate dehydrogenase (*gapdh*) was used as a nonregulated, reference gene for normalizing the expression of the verified genes (Pérez et al. 2008). qPCR was performed according to the methodology earlier described (Sadkowski et al. 2014). The results were evaluated according to the Livak method (Schmittgen and Livak 2008).

### Western blot analysis

The protein extracts obtained from the skeletal muscle tissues were lysed with RIPA buffer (50 mM Tris, pH 7.5, 150 mM NaCl, 1 mM EDTA, 1% NP-40, 0.25% Na-deoxycholate, and 1 mM PMSF) supplemented with protease inhibitor cocktail and phosphatase inhibitor cocktail (Sigma-Aldrich, USA) for 30 min at 4 °C. The lysates were cleared for 30 min at 14000 rpm, and the supernatants were collected. The concentration of protein in the lysates was determined using Bio-Rad Protein Assay Dye Reagent according to the manufacturer’s instructions (Bio-Rad Laboratories Inc., USA). The proteins (50 μg) were resolved by SDS-PAGE and transferred onto a PVDF membrane (Sigma-Aldrich, USA). For immunostaining, the membranes were blocked with 5% nonfat dry milk in TBS (20 mM Tris-HCl, 500 mM NaCl) containing 0.5% Tween 20. The membranes were then incubated with the following primary antibodies: monoclonal anti-mouse GDF-8 antibody (R&D Systems, USA) (1:100) and polyclonal goat GAPDH antibody (Santa Cruz Biotechnology, USA) (1:500). Then, the membranes were washed three times for 15 min and incubated further with appropriate secondary antibodies conjugated with an IRDye 680 or IRDye 800 CW fluorophore (LI-COR Biosciences, USA) (1:5000). An Odyssey Infrared Imaging System (LI-COR Biosciences, USA) was used to analyze the fluorescence. The scan resolution of the instrument was set at 169 μm, and the intensity at 4. The integrated optical density (IOD) was quantified using the analysis software provided with the Odyssey scanner (LI-COR Biosciences, USA) and normalized to GAPDH. Immunoblot analysis was performed on the samples obtained from four individuals of each breed (*n* = 4). For the purpose of publication, the color immunoblot images were converted into black and white in the Odyssey software.

### Statistical analysis

Statistical analysis of the microarray data was performed using the GeneSpring software (Agilent Technologies, USA). The results of qPCR and Western blot were analyzed using Prism 5.0 (GraphPad Software, USA). Statistical significance was checked using the one-way ANOVA (ANalysis Of VAriance) with Tukey post hoc test. The differences at the level of *p* ≤ 0.05, *p* ≤ 0.01, and *p* ≤ 0.001 were considered as significant and marked with one (*), two (**), and three (***) asterisks, respectively. The data are shown as means ± SEM (standard error of the mean).

### Functional analysis

The results were subjected to ontological analysis using the following online available databases: Panther Classification System (http://www.pantherdb.org) with a statistical overrepresentation test with Bonferroni correction; DAVID (Database of Annotation, Visualization, and Integrated Discovery) (https://david.ncifcrf.gov/), and Pathway Studio Web (Elsevier, Netherlands). In addition, during the development of data from microarrays, numerous internet databases, grouped in NCBI, such as GenBank, OMIM, PubMed, and iHOP, were used.

## Results

### Transcriptomic analysis

**Fig. 1:**
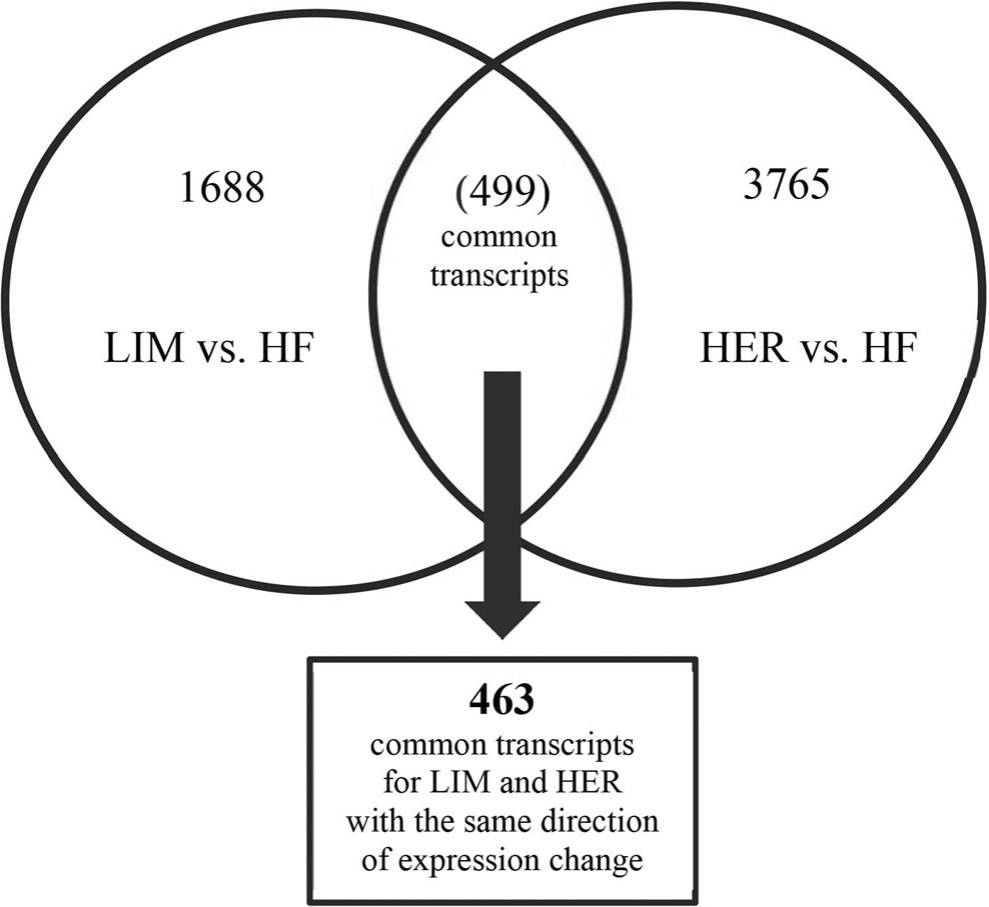
Number of transcripts identified in the comparison of semitendinosus muscle tissue in 15-month-old bulls from beef breeds (LIM and HER) with semitendinosus muscle transcriptomes of bulls from dairy breed (HF); *p* ≤ 0.05, FC ≥ 1.3, *n* = 4 for each breed

Four hundred ninety-nine common transcripts were identified for individuals of LIM and HER breeds, relative to individuals of the HF breed, in which expression differences were statistically significant (Fig. 1). Four hundred sixty-three common transcripts were selected for further analyses, whose expression change occurred in the same direction in both beef breeds. It was assumed that the selection criterion will enable identification of the putative genes regulating the process of muscle organ development, as well as influencing greater growths of muscle mass in bulls of beef breeds than in dairy breed. Among the 463 identified transcripts, 236 demonstrated elevated, and 227 reduced expression in the case of beef breeds than in dairy breed. Sequences, to which the manufacturer of microarray did not assign gene name, were compared with the NCBI Blastn Nucleotide base in order to assign gene name. Sequences not showing 100% complementarity with bovine or mammalian mRNA were excluded from further analyses. As a result of further ontological analyses, all common, non-duplicated genes (454) with the same direction of expression change in beef breeds (differentially expressed genes—DEGs).

**Fig. 2:**
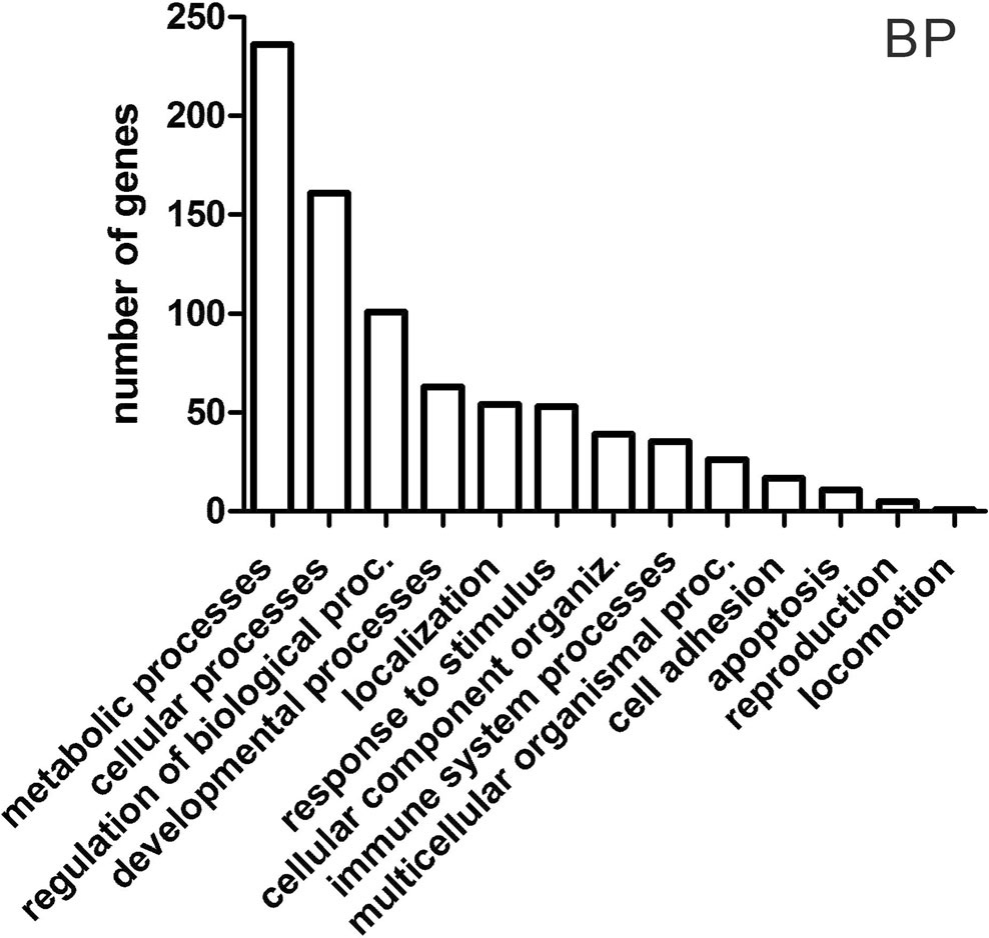
Functional classification of differentially expressed genes in terms of their participation in biological processes—BP (Panther)

Using the Panther software, classification of the DEGs (454) was performed in terms of biological processes, in which products of expression of these genes might be involved. Ontological analysis demonstrated their relationship with 13 biological processes, including the following: metabolic processes (236 genes), cellular processes (161), regulation of biological processes (101), developmental processes (63), localization (54), response to stimulus (53), cellular component organization or biogenesis (39), immune system processes (35), multicellular organismal processes (26), cell adhesion (17), apoptosis (11), reproduction (5), and locomotion (1) (Fig. 2). In the further ontological analysis, the genes, which were assigned by the PANTHER 7.0 software to metabolic processes and developmental processes, were taken into account.

#### Metabolic processes

Among metabolic processes, the greatest gene cluster was assigned with primary metabolic process (195 genes), followed by phosphate-containing compound metabolic process (31), nitrogen compound metabolic process (27), biosynthetic process (17), catabolic process (11), generation of precursor metabolites and energy (5), coenzyme metabolic process (3), sulfur compound metabolic process (2), and vitamin metabolic process (1).

**Fig. 3:**
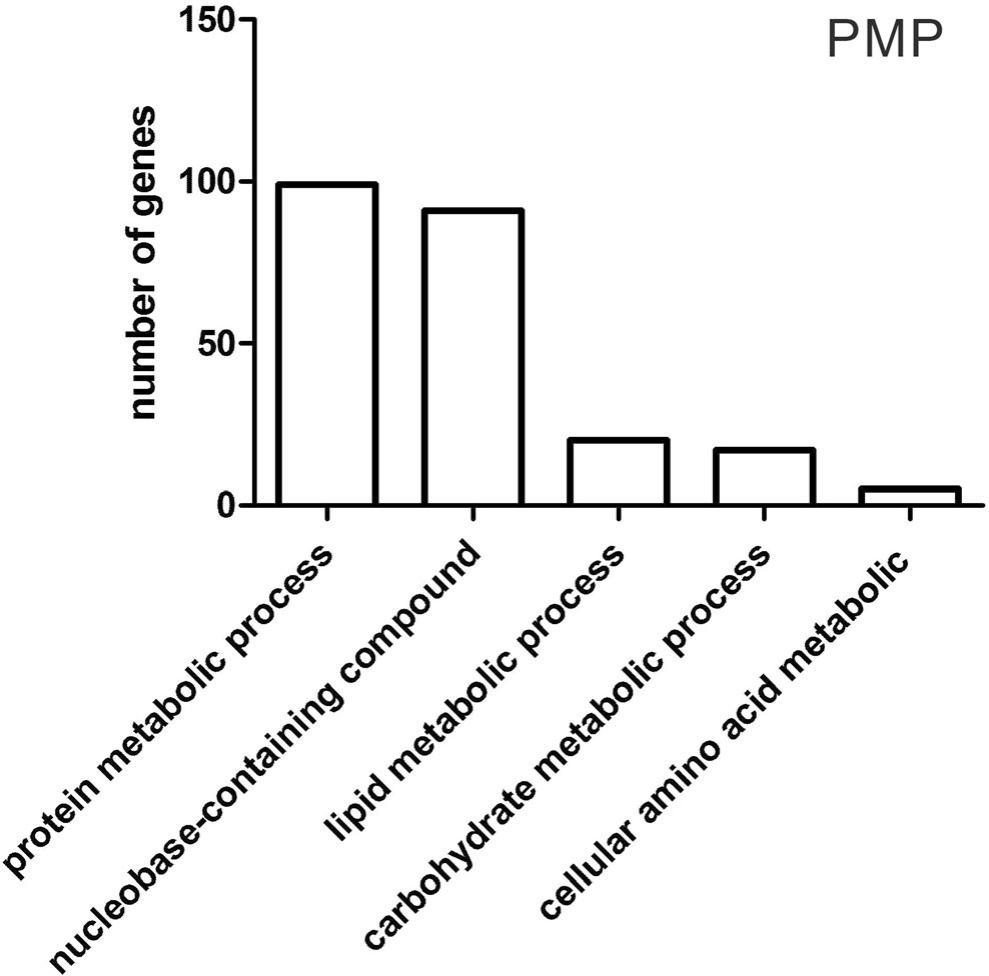
Functional classification of differentially expressed genes in terms of their participation in primary metabolic processes—PMP (Panther)

Further functional classification of DEGs demonstrated that among 195 genes involved in primary metabolic processes, the highest share was exhibited by the genes involved in protein metabolic process (99), nucleobase-containing compound metabolic process (91), lipid metabolic process (20) carbohydrate metabolic process (17), and cellular amino acid metabolic process (5) (Fig. 3). The multiplicity of the change with directions of expression of 99 DEGs associated with protein metabolic process is shown in Supplementary Table 2.

**Fig. 4:**
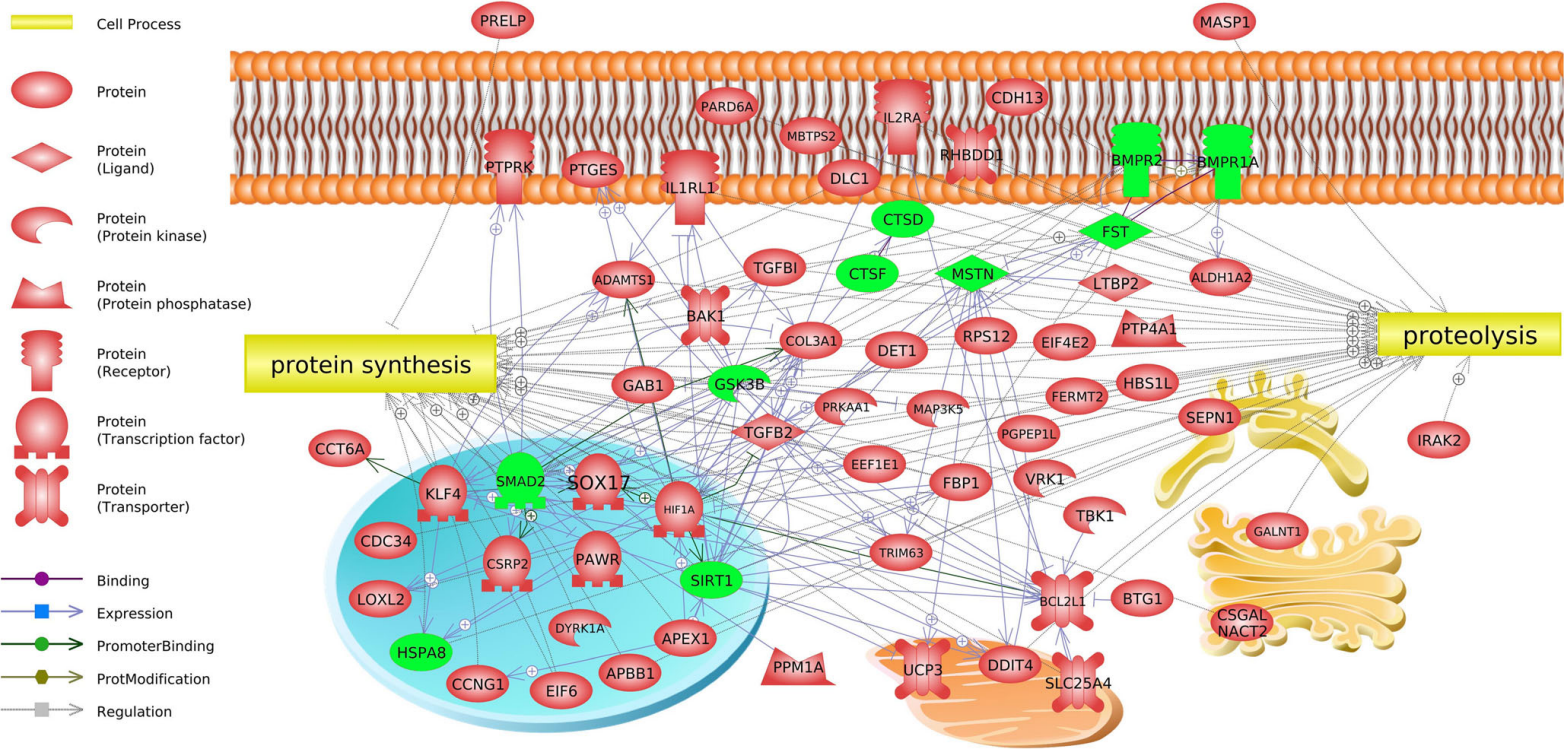
Network of intergenic interactions of differentially expressed genes and their classification to processes associated with protein metabolism (Pathway Studio). The discussed genes are marked in green

Using the Pathway Studio software, a network of relationships was performed for the differentially expressed genes assigned to the processes associated with protein metabolic process. This enabled a visualization of interaction between DEGs and additionally demonstrated the affiliation of genes to two major processes associated with protein metabolic process: protein synthesis (36 genes) and proteolysis (33 genes) (Fig. 4, Supplementary Table 3).

#### Developmental processes

**Fig. 5:**
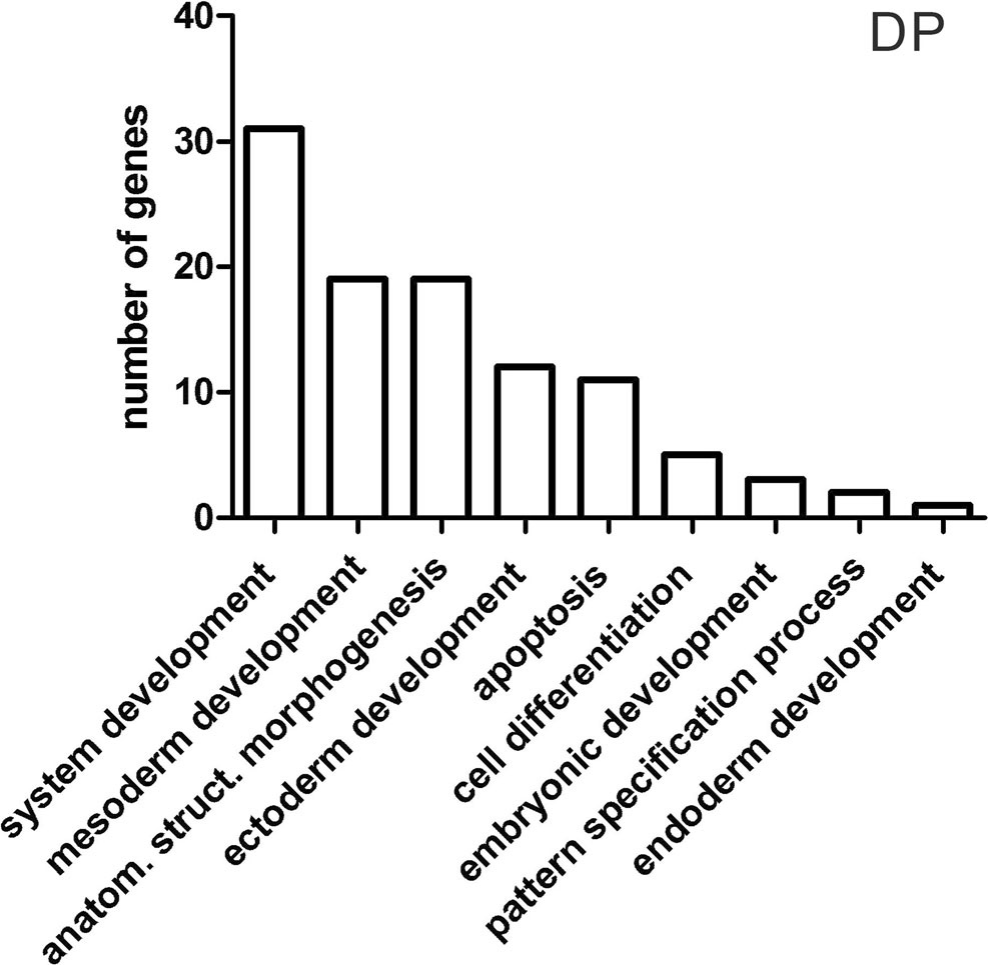
Functional classification of differentially expressed genes in terms of their participation in developmental processes—DP (Panther)

Functional analysis of genes performed using the Panther software demonstrated that among 63 genes involved in developmental processes, the highest share was demonstrated by the genes participating in the following processes: system development (31 genes), mesoderm development (19), anatomical structure morphogenesis (19), ectoderm development (12), apoptosis (11), cell differentiation (5), embryonic development (3), pattern specification process (2), and endoderm development (1) (Fig. 5). The process associated with muscle organ development was represented by 8 DEGs; the multiplicity of change with the directions of expression changes is presented in Supplementary Table 4.

In addition, an ontological analysis was performed using the Pathway Studio software, which assigned the DEGs with the same direction of expression change (454 genes) to 473 biological processes (*p* ≤ 0.05). Supplementary Table 5 demonstrates selected biological processes associated with skeletal muscle organ development and additionally processes occurring in smooth and cardiac muscle. The above analysis demonstrated that these processes are represented by 58 DEGs whose fold change with the direction of expression changes is presented in Supplementary Table 6.

### Verification with the use of qPCR

**Fig. 6:**
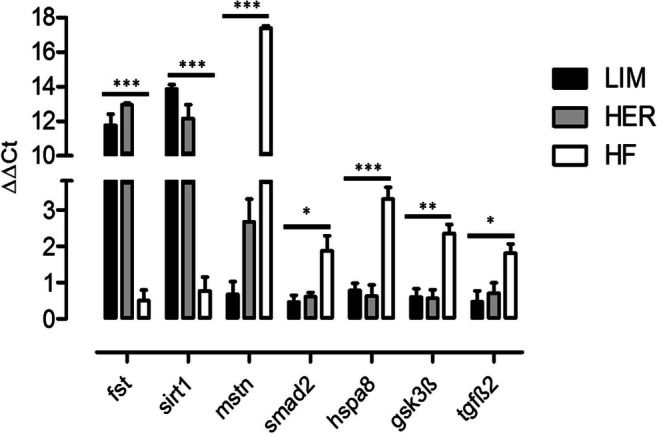
Verification of transcriptomic analysis performed using the real-time qPCR technique. Seven genes were selected for analysis, associated with muscle tissue development and protein metabolism processes. The *gapdh* gene was used as reference. Statistical analysis of the obtained results was carried out using the GraphPad Prism 5 (GraphPad Software Inc., USA) software utilizing one-way ANOVA Tukey’s multiple range test. The results have been presented as mean ± standard error (SEM) and were marked as statistically significant *for *p* < 0.05; **for *p* < 0.01; and ***for *p* < 0.001. Difference significance at the level of *p* ≤ 0.05 was considered significant (*n* = 4 for each breed)

In order to verify the results of transcriptomic analysis, validation of gene expression with the use of qPCR technique has been performed. For the purposes of result verification, seven genes were selected, demonstrating similar changes of expression in both beef breeds (LIM and HER) compared with typical dairy breed (HF), associated with the processes of muscle tissue development and protein metabolism processes. Results of verification with real-time qPCR are presented in the form of block diagrams. The level of mRNA expression is presented as ΔΔCt (Fig. 6). The qPCR demonstrated higher level of expression of two genes (*fst*, *sirt1*) and lower level of expression of 5 genes (*mstn*, *smad2*, *hspa8*, *gsk3β*, *tgf-β2*) in semitendinosus muscle of beef breeds (LIM and HER), compared with the dairy breed (HF). These results confirm the results obtained with microarray analysis and exhibited the same direction of expression change as in the microarray experiment.

### Analysis of myostatin protein level

**Fig. 7:**
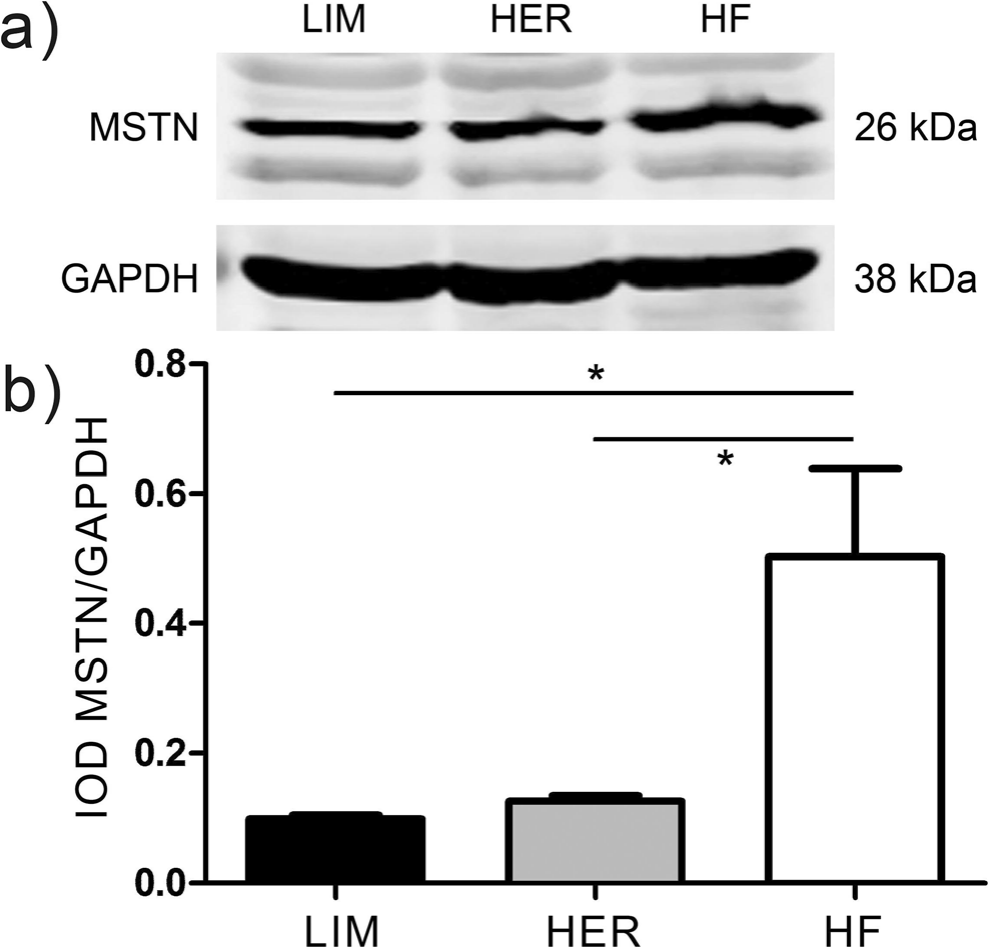
Western blot analysis of myostatin expression level in mature tissue of semitendinosus muscle (*m. semitendinosus*) originating from three tested breeds. **a** Western blot analysis demonstrating expression level of active form of myostatin (26 kDa). GAPDH (38 kDa) was used as reference protein. **b** Values of integrated optical density (IOD) obtained for individual stripes, expressed in relative values, determined as a ratio of the obtained value of IOD for myostatin to IOD value for corresponding GAPDH stripe. Statistical significance of MSTN expression differences was calculated using one-way ANOVA Tukey’s multiple range test. The results were presented as mean ± SEM, calculated with the GraphPad software (GraphPad Software Inc., USA). Statistical significance was determined*for *p* ≤ 0.05, (*n* = 4 for each breed)

Analysis of gene expression with microarray and qPCR shows that myostatin (MSTN), the key protein inhibiting the process of myogenesis, may be one of the factors influencing differences in the musculature of bulls from beef breeds (LIM, HER), compared with bulls of the dairy breed (HF) (Grobet et al. 1997; Kambadur et al. 1997; Sadkowski et al. 2008). Western-blot analysis demonstrated statistically significant (*p* ≤ 0.05) difference in the expression of MSTN in bulls from LIM and HER breeds compared with HF breed bulls. Expression of the active form of myostatin (26 kDa) in the semitendinosus muscle tissue was lower in beef breeds compared with dairy breed (Fig. 7).

## Discussion

Intensified synthesis of proteins and their reduced proteolysis is responsible for promotion of muscle mass growth in a mature muscle tissue (Sartori et al. 2009). The present study demonstrated significant differences in expression of genes associated with protein synthesis and proteolysis (Supplementary Tables 2, 6), playing or capable of playing significant role in the process of growth and development of muscle tissue, and thus influencing obtaining higher muscle tissue gains in bulls from beef breeds.

One of the key genes involved in the processes of growth and development of muscle tissue is follistatin (*fst*) and myostatin (*mstn*). The present study demonstrated higher expression of follistatin and lower expression of myostatin in beef bulls (LIM and HER) compared with bulls from dairy breed (HF) (Supplementary Table 2), which was also confirmed using qPCR (Fig. 6). In addition, lower myostatin protein level was observed in beef breeds compared with dairy breed (Fig. 7). The Pathway Studio software assigned *fst* and *mstn* to process associated with protein synthesis and proteolysis (Fig. 4, Supplementary Table 3) and processes associated with muscle organ development (Supplementary Table 5). It is known that follistatin is a strong antagonist of myostatin and it has stimulatory effect on the increase of muscle mass (Zheng et al. 2017). The role of follistatin as inhibitor of myostatin consists in the capacity to bind myostatin, thus disabling its binding with activin type II receptor (ActRII), preventing reduction of expression of genes responsible for the growth and differentiation of myogenic cells (Lee 2007; Lee et al. 2012; Lie et al. 2015; Formicola et al. 2018).

Matzuk et al. (1995) determined that mice deprived of follistatin demonstrate mortality after birth, associated with insufficient muscle development and muscle anomalies. Similarly, Lee et al. (2010) demonstrated that mice with fst ± mutation exhibit reduced muscle mass. On the other hand, Lee and McPherron (2001) demonstrated that mice with overexpression of *fst* are characterized by elevated muscle mass, the so called double-muscled phenotype, which is also observed in mice with myostatin knock-out. Moreover, Chang et al. (2017) demonstrated in transgenic pigs that overexpression of *fst* considerably increased skeletal muscle mass and reduced fat accumulation. Furthermore, follistatin demonstrates inhibiting action for the activity of activins and BMP2, representatives of the TGFβ superfamily other than myostatin (Zheng et al. 2017).

Lower *mstn* expression on both transcript level (Fig. 6, Supplementary Tables 2, 6) as well as at protein level (Fig. 7) in cattle of LIM and HER breed, may also contribute to stimulation of muscle growth and development process and to attain greater muscle mass growths by beef breeds. Results of numerous in vitro studies have demonstrated that myostatin inhibits the process of proliferation and differentiation of muscle cells with maturation of skeletal muscles, preventing their excessive hypertrophy (Kocamis et al. 2001; Chargé and Rudnicki 2004; Sato et al. 2006; Lee et al. 2016; Ahmad et al. 2017). Moreover, Taylor et al. (2001) and Deng et al. (2017) observed under in vitro conditions that in myoblast and myotube cultures, myostatin has inhibitory effect on the process of protein synthesis. Inhibition of myostatin activity may influence the process of protein synthesis via various mechanisms. One of such mechanisms is intensification of fusion of satellite cells with the growing muscle, and thus supply of new cell nuclei increasing the amount of nuclear DNA of the fiber, resulting in increased protein synthesis, observed in muscle fiber hypertrophy (Welle et al. 2009; Pallafacchina et al. 2013; Goh and Millay 2017). Another mechanism influencing intensification of protein synthesis process, after inhibition of myostatin activity, is the increase of expression of genes encoding transcription initiation and mRNA elongation regulating factors and genes encoding proteins involved in translation (Welle et al. 2009). These factors are, among others: paired-box transcription factor Pax3 (Pax3), myoblast determination protein 1 (MyoD), myogenic factor 5 (Myf5), and myogenin (Myog) (Amthor et al. 2002; Langley et al. 2002; Bakkar et al. 2005; McFarlane et al. 2006;), as well as ribosomal protein S6 kinase (S6K), ribosomal protein S6 (rpS6), translation initiation factor 5-subunit A (Eif5a), and ribosomal protein S27-like (Rps27l) (Welle et al. 2009; Rodriguez et al. 2014); however, aforementioned factors were not detected as differentially expressed genes in the presented study. In many animal species, myostatin is responsible for reducing the muscle mass by both functional impairment of satellite cells as well as via reducing protein synthesis (Taylor et al. 2001; McCroskery et al. 2003; Suryawan et al. 2006; Welle et al. 2006; McFarland et al. 2007; Dasarathy et al. 2011). Periyalwar and Dasarathy (2012) and Wang et al. (2015) demonstrated that myostatin also intensifies the process of protein proteolysis, contributing to muscle dystrophy. Inhibition of myostatin activity further has potential benefits for the breeders of farm animals, as it may increase muscle mass growths of cattle (hypertrophy of muscle tissue) and percentage content of lean meat in the carcass (Huang et al. 2011; Camporez et al. 2016).

*Smad2* (*SMAD family member 2*) is another gene identified using the microarray technique, whose expression level was lower in bulls of beef breeds than in the dairy breed (Fig. 6, Supplementary Table 6). Using the Pathway Studio software, *smad2* was classified in processes associated with muscle growth (Supplementary Table 5) and process associated with protein synthesis and proteolysis (Fig. 4, Supplementary Table 3). Smad2 intensifies proteolysis process of muscle-specific proteins (Sartori et al. 2014). The smad2/smad3 proteins are activated by myostatin and activin A. Both ligands bind with activin type II receptors (ActRIIB and ActRIIA), followed by activin type I receptors (ALK5 or ALK4) to activate smad2/smad3, leading to proteolytic destruction of skeletal muscle proteins and resulting in atrophy of muscle fibers (Dutt et al. 2015). Similarly, Chen et al. (2014) observed that as a result of activation of smad2/smad3 transcription factors by myostatin and activin A, the protein degradation process occurs, and their synthesis is inhibited, resulting in skeletal muscle atrophy in mice. Tando et al. (2016) demonstrated Smad 2/3 protein accumulation leading to muscle atrophy, also in mouse model. It is possible that lower *smad2* expression level in LIM and HER bulls as contrasted with HF breed has a positive impact on the development of skeletal muscles and promotes obtaining greater muscle mass gains in these breeds, as a result of i.a. inhibition of protein degradation and stimulation of their synthesis process, which favors hypertrophy of muscle tissue. Moreover, lower *smad2* expression level may also stem from the lower expression of myostatin in the muscles of beef breed bulls, both at mRNA and protein level (Figs. 6 and 7, Supplementary Table 2).

The present study has further demonstrated lowered expression of *bmpr1a* (bone morphogenetic protein receptor, type IA) and *bmpr2* (bone morphogenetic protein receptor, type II) in mature 15-month-old muscle tissue in LIM and HER breeds compared with HF breed (Supplementary Tables 2, 6). In the Panther database, *g*enes *bmpr1a* and *bmpr2* were classified to the protein metabolism process (Supplementary Table 2), whereas the Pathway Studio software assigned them to processes associated with muscle organ development (Supplementary Table 5). Visualization of interaction between the above genes is presented in Fig. 4. Suryawan et al. (2006) showed negative impact of bmp2 and bmp7 on the process of muscle protein synthesis. The mentioned genes constitute negative regulators of skeletal muscle growth classified in the TGFβ superfamily, acting through specific membrane receptor, BMPR1A. Dörpholz et al. (2017) demonstrated that treatment of cells with BMP2 efficiently inhibited differentiation of myoblasts into myotubes. Moreover, the BMP2 stimulation almost entirely inhibited the expression of myogenic markers. Authors suggest that activation of the BMP signal pathway impeded the myogenic differential. Similarly, Friedrichs et al. (2011) and Ono et al. (2011) demonstrated that BMP inhibits differentiation of primary satellite cells and favors proliferation of precursor cells of muscles. Moreover, Amthor et al. (2004) demonstrated that follistatin reverses the inhibitory effect of bmp2 and bmp7 on the development of skeletal muscles via blockage of their receptors activation. It is likely that the higher *fst* expression of beef breed bulls may also influence reduction of *bmpr1a* and *bmpr2* expression, and thus limit the negative impact of factors acting through these receptors on the process of muscle protein synthesis.

Another gene, the expression level of which was lower in the semitendinosus muscle of beef breed individuals compared with dairy breed, is *hspa8* (heat shock 70 kDa protein 8) (Fig. 6, Supplementary Table 2), also known as *hsc70* or *hsp73*. Functional analysis (Panther) assigned this gene to protein metabolism process (Supplementary Table 2), whereas the Pathway Studio software classified it to the protein synthesis and proteolysis process (Fig. 4, Supplementary Table 3). Heat shock proteins are responsible for the intensification of the protein degradation process (Mambula et al. 2007; Liu et al. 2012). HSPA8 is an isoform of the Hsp70/Hsc70 family (Fernández-Fernández et al. 2017) and it plays significant role in cell protein degradation, in particular through its influence on the ubiquitin-proteasome pathway (Stricher et al. 2013). Fernández-Fernández et al. (2017) further demonstrated that cellular proteins are degraded by signal pathways independent of Hsp-70. HSPA8 contributes to proteasomal protein degradation (Valek et al. 2019). It is possible that reduced level of *hspa8* expression in LIM and HER breeds than in HF breed may contribute to inhibition of muscle protein degradation process.

Comparison of transcriptomes of beef breeds with dairy breed of cattle demonstrated lower level of *gsk3β* expression in beef breeds (Fig. 6, Supplementary Tables 2, 6). Functional analysis linked this gene with protein metabolism (Supplementary Table 2), both their synthesis and proteolysis (Fig. 4, Supplementary Table 3) and muscle organ development (Supplementary Table 5). Numerous previous studies have demonstrated that *gsk3β* inhibits the process of protein synthesis with concomitant stimulation of their degradation. The *gsk3β* overexpression restricts protein synthesis and expression of genes responsible for synthesis of muscle proteins, and thus hypertrophy of muscle fibers (Mohamed et al. 2010). Moreover, it has been presented that activation of the GSK3β signal pathway leads to elevated expression of proteolysis markers, myogenesis disruption, and to muscle loss (Shen et al. 2017). However, inhibition of GSK3β prevents muscle atrophy in mice (Shen et al. 2017) and guinea pigs (Verhees et al. 2013), and it further prevents development of myotonic dystrophy type 1 (DM1), eventually leading to skeletal muscle being characterized by higher fiber density, and normal fiber size (Wei et al. 2018). Other researchers also observed that GSK3β is a negative regulator of skeletal muscle growth, and its insufficiency stimulates myogenic differentiation and myotube formation (van der Velden et al. 2008; Ma et al. 2014).

The conducted transcriptomic analysis further indicated higher level of *sirt1* (sirtuin 1) expression in the muscle of LIM and HER bulls (Fig. 6, Supplementary Table 6). This gene was classified via functional analysis as participating in muscle organ development processes (Supplementary Table 5) and protein synthesis and proteolysis (Fig. 4, Supplementary Table 3). Lee and Goldberg (2013) demonstrated that *sirt1* overexpression contributes to hypertrophy of mice muscles and it inhibits their atrophy through blocking proteolysis. The authors showed that *sirt1* activation stimulates muscle growth process in the post-natal period and inhibits expression of genes associated with muscle protein proteolysis, such as MuRF1 (known as *trim63*) and atrogin 1. The authors observed that as a result of *sirt1* overexpression, reduction of the protein degradation degree occurs and their synthesis is intensified, which resulted in increased muscle mass. Koltai et al. (2017) further observed that hypertrophy of muscles in rats was associated with elevated protein content and SIRT1 activity. On the other hand, reduced expression or inactivation of SIRT1 impedes activation of satellite cells and regeneration of skeletal muscles (Tang and Rando 2014; Ryall et al. 2015).

Other genes, which may constitute important regulators of growth and development of skeletal muscles, and whose expression level was higher in beef breeds than in dairy breed, are *ctsd* (cathepsin D) and *ctsf* (cathepsin F) (Supplementary Table 2). The Pathway Studio software assigned *ctsd* to the protein synthesis and proteolysis process (Fig. 4, Supplementary Table 3). It is known that the loss of *ctsd* function has negative impact on the normal development and function of numerous tissues, including epithelium, nervous, and muscle tissue, as well as organs such as the brain, eye, heart, and intestines (Follo et al. 2011).

Higher expression in the muscle tissue of LIM and HER bulls than in HF breed was demonstrated also for *fhl2* (four and a half LIM domains 2) and *fhl3* (four and a half LIM domains 3) genes, which are associated with the development of muscle organ (Supplementary Table 4). FHL2 and FHL3 belong to the FHL protein family (four and a half LIM domain proteins), which play the putative role in the development and growth control of animals and differentiation of cells. *Fhl2* and *fhl3* are subject to expression during myocardium and skeletal muscle development, both in prenatal and post-natal period; however, their expression in mature muscles is predominant and it indicates their significant role during the development and growth of muscles (Chu et al. 2000). Additionally, Martin et al. (2002) observed that C2C12 line cells exhibiting *fhl2* overexpression were characterized by elevated differentiation degree, accelerated formation of myotubes, and elevated expression of proteins characteristic of muscle fibers, suggesting that *fhl2* promotes myogenesis process.

## Conclusions

Comparison of transcriptomic profiles of fully differentiated skeletal muscle originating from 15-month-old bulls from various breeds with different performance enabled formulation of the following conclusions. Greater growths of muscle mass in bulls from beef breeds (LIM, HER) than in the dairy breed (HF) stem from the differences in protein transformations and are associated with elevated expression of genes stimulating synthesis and inhibiting degradation of proteins (*fst*, *sirt1*) and decreased expression of genes responsible for inhibition of synthesis and intensification of protein degradation (*mstn*, *smad2*, *hspa8*, *gsk3β*). Increased level of follistation expression and decreased myostatin expression level in skeletal muscles of beef breed bulls indicate the role of follistatin as antagonist of myostatin in the regulation of myogenesis, and it may play a significant role in stimulating development and growth of muscles in these breeds, and as a consequence greater gains of muscle mass. Moreover, the faster growth and development of muscle tissue in beef bulls can be associated with the elevated expression of *fhl2* and *fhl3* genes encoding FHL proteins (four and a half LIM domain proteins), playing putative role in the process of skeletal muscle differentiation including formation of myotubes and expression of proteins characteristic of muscle fibers. The genes identified in this study, involved in the regulation processes of growth and development of muscle tissue and protein metabolism processes in the tested cattle breeds, may be responsible for greater muscle mass gains in bulls from beef breeds and may constitute a new cattle selection tool towards highly efficient meat production, which may be of considerable economic importance.

## Electronic supplementary material

ESM 1 (DOCX 73 kb)
