# Supplementary material for: Transcriptomic profile of semitendinosus muscle of bulls of different breed and performance
Source: J Appl Genet. 2020 Aug 26;61(4):581–92. doi: 10.1007/s13353-020-00577-1 (PMC7652804; doi:10.1007/s13353-020-00577-1)
Supplement: Supplementary file 1 — (DOCX 73 kb) [file 13353_2020_577_MOESM1_ESM.docx]

**Supplementary Table 1** Primers used for Real-time qPCR

| **Gene**  **symbol** | **Accession number** | **Forward primer** | **Reverse primer** | **Annealing temp. (°C)** | **PCR product length** |
| --- | --- | --- | --- | --- | --- |
| *fst* | NM_175801 | 5'AGTCTGAGGAGCCTGTCTGTG3' | 5'CCTCCTCTTCCTCGGTGTCTT3' | 63 | 181 |
| *gsk3β* | NM_001101310 | 5'AAATACCACAGCAGCCTCAGA3' | 5'CTTTCCAATCGTGACCAGTGT3' | 60 | 167 |
| *hspa8* | NM_174345 | 5'AGACTTGCTGCTGTTGGATGT3' | 5'GCAGGTTGTTATCCTTGGTCA3' | 60 | 200 |
| *mstn* | NM_001001525 | 5'CACTGGTGTGGCAAGTTGT3' | 5'CTTCTGCTCGCTGTTCTCA3' | 58 | 218 |
| *sirt1* | NM_001192980 | 5'TGTTGGGAGGACTTGGTGTA3' | 5'ATGTTGGGGAAAGGTCTGAG3' | 58 | 250 |
| *smad2* | NM_001046218 | 5'AGCAGAATACCGAAGGCAGA3' | 5'TGATGGGACTTGATTGGTGA3' | 58 | 163 |
| *tgf-β2* | NM_001113252 | 5'ACATCATCCCCAACAAAAGCG3' | 5'TCAAGTCTGTAGGAGGGCAAC3' | 60 | 205 |
| *gapdh* | NM_001034034 | 5'GTTTGTGATGGGCGTGAACC3' | 5'GTCTTCTGGGTGGCAGTGAT3' | 58 | 198 |

**Supplementary Table 2** List of differentially expressed genes associated with protein metabolism processes (*Panther;* FC≥1.3, FC - Fold change; p≤0.05, n=4 for each breed; R - Regulation, direction of expression change was marked with arrow up (increase of expression) and arrow down (expression decrease)

| **No.** | **Gene symbol** | **Description** | **LIM vs. HF** | | | **HER vs. HF** | | |
| --- | --- | --- | --- | --- | --- | --- | --- | --- |
|  |  |  | **FC** | **R** | **p-value** | **FC** | **R** | **p-value** |
| 1 | *cfb* | Bos taurus complement factor B (CFB), mRNA [NM_001040526] | 6,37 | ↑ | 9,10E-03 | 4,92 | ↑ | 1,03E-02 |
| 2 | *slc25a33* | Bos taurus solute carrier family 25, member 33 (SLC25A33), mRNA. [Source:RefSeq mRNA;Acc:NM_001076002] [ENSBTAT00000004127] | 5,35 | ↑ | 3,09E-03 | 1,90 | ↑ | 1,47E-02 |
| 3 | *trib1* | Bos taurus tribbles homolog 1 (Drosophila) (TRIB1), mRNA [NM_001101105] | 3,94 | ↑ | 2,36E-03 | 2,31 | ↑ | 1,70E-02 |
| 4 | *c3* | Bos taurus complement component 3 (C3), mRNA [NM_001040469] | 3,57 | ↑ | 2,71E-02 | 5,52 | ↑ | 3,16E-03 |
| 5 | *rhbdd1* | Bos taurus rhomboid domain containing 1 (RHBDD1), mRNA [NM_001077995] | 2,83 | ↑ | 1,52E-02 | 1,39 | ↑ | 2,45E-02 |
| 6 | *adamts1* | Bos taurus ADAM metallopeptidase with thrombospondin type 1 motif, 1 (ADAMTS1), mRNA [NM_001101080] | 2,81 | ↑ | 1,16E-02 | 1,32 | ↑ | 7,44E-04 |
| 7 | *ptprk* | Bos taurus protein tyrosine phosphatase, receptor type, K (PTPRK), mRNA [NM_001191537] | 2,51 | ↑ | 4,68E-02 | 2,84 | ↑ | 1,74E-02 |
| 8 | *cfd* | Bos taurus complement factor D (adipsin) (CFD), mRNA [NM_001034255] | 2,40 | ↑ | 1,66E-02 | 2,74 | ↑ | 1,43E-03 |
| 9 | *itih4* | Bos taurus inter-alpha-trypsin inhibitor heavy chain family, member 4 (ITIH4), mRNA [NM_001015590] | 2,39 | ↑ | 1,45E-03 | 2,31 | ↑ | 6,32E-03 |
| 10 | *masp1* | Bos taurus mannan-binding lectin serine peptidase 1 (C4/C2 activating component of Ra-reactive factor) (MASP1), mRNA [NM_001076968] | 2,34 | ↑ | 6,70E-03 | 2,15 | ↑ | 3,62E-04 |
| 11 | *c4a* | Bos taurus complement component 4A (C4A), mRNA [NM_001166485] | 2,30 | ↑ | 2,92E-02 | 5,92 | ↑ | 1,12E-03 |
| 12 | *ctsf* | Bos taurus cathepsin F (CTSF), mRNA [NM_001075416] | 2,24 | ↑ | 2,87E-02 | 2,37 | ↑ | 9,68E-03 |
| 13 | *wnk4* | WNK lysine deficient protein kinase 4 [Source:HGNC Symbol;Acc:14544] [ENSBTAT00000026519] | 2,22 | ↑ | 1,74E-03 | 1,73 | ↑ | 3,41E-03 |
| 14 | *pcolce2* | Bos taurus procollagen C-endopeptidase enhancer 2 (PCOLCE2), mRNA [NM_001075629] | 2,20 | ↑ | 1,20E-02 | 4,20 | ↑ | 1,86E-03 |
| 15 | *fst* | Bos taurus follistatin (FST), mRNA [NM_175801] | 2,18 | ↑ | 3,36E-02 | 2,48 | ↑ | 2,40E-02 |
| 16 | *trim63* | Bos taurus tripartite motif containing 63 (TRIM63), mRNA [NM_001046295] | 2,13 | ↑ | 3,12E-02 | 2,55 | ↑ | 2,81E-02 |
| 17 | *adamtsl5* | PREDICTED: Bos taurus KIAA2031 protein-like (LOC100337439), mRNA [XM_003585465] | 2,06 | ↑ | 1,68E-03 | 1,83 | ↑ | 3,39E-02 |
| 18 | *map3k5* | Bos taurus mitogen-activated protein kinase kinase kinase 5 (MAP3K5), mRNA [NM_001144081] | 2,05 | ↑ | 7,56E-03 | 1,99 | ↑ | 4,45E-02 |
| 19 | *tpst1* | Bos taurus tyrosylprotein sulfotransferase 1 (TPST1), mRNA [NM_001077912] | 1,97 | ↑ | 1,30E-03 | 1,71 | ↑ | 1,07E-02 |
| 20 | *tgfbr3* | PREDICTED: Bos taurus transforming growth factor, beta receptor III (TGFBR3), mRNA [XM_001253071] | 1,96 | ↑ | 6,83E-03 | 1,97 | ↑ | 2,35E-02 |
| 21 | *uhrf2* | Bos taurus ubiquitin-like with PHD and ring finger domains 2 (UHRF2), mRNA [NM_001102270] | 1,93 | ↑ | 2,87E-02 | 1,37 | ↑ | 4,61E-03 |
| 22 | *cdc42ep4* | Bos taurus CDC42 effector protein (Rho GTPase binding) 4 (CDC42EP4), mRNA [NM_001046471] | 1,92 | ↑ | 1,49E-02 | 1,81 | ↑ | 4,68E-02 |
| 23 | *dstn* | Bos taurus destrin (actin depolymerizing factor) (DSTN), mRNA [NM_001015586] | 1,88 | ↑ | 7,02E-03 | 1,44 | ↑ | 1,08E-02 |
| 24 | *usp53* | ubiquitin specific peptidase 53 [Source:HGNC Symbol;Acc:29255] [ENSBTAT00000049054] | 1,79 | ↑ | 9,63E-03 | 1,73 | ↑ | 2,64E-02 |
| 25 | *vrk1* | Bos taurus vaccinia related kinase 1 (VRK1), mRNA [NM_001038224] | 1,72 | ↑ | 4,52E-02 | 1,45 | ↑ | 3,05E-02 |
| 26 | *prelp* | proline/arginine-rich end leucine-rich repeat protein [Source:HGNC Symbol;Acc:9357] [ENSBTAT00000023709] | 1,67 | ↑ | 2,72E-03 | 1,99 | ↑ | 1,84E-03 |
| 27 | *dtd1* | Bos taurus D-tyrosyl-tRNA deacylase 1 homolog (S, cerevisiae) (DTD1), mRNA [NM_001038104] | 1,67 | ↑ | 8,24E-03 | 1,58 | ↑ | 5,09E-03 |
| 28 | *zbtb9* | Bos taurus zinc finger and BTB domain containing 9 (ZBTB9), mRNA [NM_001191462] | 1,66 | ↑ | 1,96E-02 | 1,58 | ↑ | 4,93E-02 |
| 29 | *kat5* | Bos taurus K(lysine) acetyltransferase 5 (KAT5), mRNA [NM_001192066] | 1,66 | ↑ | 2,33E-02 | 1,97 | ↑ | 3,97E-03 |
| 30 | *pdk1* | Bos taurus pyruvate dehydrogenase kinase, isozyme 1 (PDK1), mRNA [NM_001205957] | 1,64 | ↑ | 1,84E-02 | 1,36 | ↑ | 9,72E-03 |
| 31 | *adprhl2* | Bos taurus ADP-ribosylhydrolase like 2 (ADPRHL2), mRNA [NM_001035340] | 1,61 | ↑ | 1,70E-02 | 1,48 | ↑ | 2,07E-03 |
| 32 | *sco1* | Bos taurus SCO cytochrome oxidase deficient homolog 1 (yeast) (SCO1), mRNA [NM_001080243] | 1,58 | ↑ | 2,03E-02 | 1,33 | ↑ | 2,55E-02 |
| 33 | *pacsin2* | Bos taurus protein kinase C and casein kinase substrate in neurons 2 (PACSIN2), mRNA [NM_001046468] | 1,51 | ↑ | 1,42E-02 | 1,57 | ↑ | 3,27E-02 |
| 34 | *pgpep1l* | PREDICTED: Bos taurus pyroglutamyl-peptidase I-like (PGPEP1L), transcript variant X3, mRNA [XM_870038] | 1,49 | ↑ | 2,92E-02 | 1,79 | ↑ | 3,07E-02 |
| 35 | *ptpn13* | Bos taurus protein tyrosine phosphatase, non-receptor type 13 (APO-1/CD95 (Fas)-associated phosphatase) (PTPN13), mRNA [NM_174590] | 1,47 | ↑ | 5,59E-03 | 1,91 | ↑ | 8,65E-03 |
| 36 | *rnf114* | Bos taurus ring finger protein 114 (RNF114), mRNA [NM_001024531] | 1,45 | ↑ | 3,19E-02 | 1,96 | ↑ | 2,21E-02 |
| 37 | *ptpn18* | Bos taurus protein tyrosine phosphatase, non-receptor type 18 (brain-derived) (PTPN18), mRNA [NM_001078069] | 1,43 | ↑ | 4,25E-05 | 1,45 | ↑ | 3,26E-02 |
| 38 | *rps20* | Bos taurus ribosomal protein S20 (RPS20), mRNA [NM_001034438] | 1,41 | ↑ | 1,71E-02 | 1,37 | ↑ | 2,88E-03 |
| 39 | *eif6* | Bos taurus eukaryotic translation initiation factor 6 (EIF6), mRNA [NM_174830] | 1,39 | ↑ | 2,54E-02 | 1,48 | ↑ | 2,20E-02 |
| 40 | *ctsd* | Bos taurus cathepsin D (CTSD), mRNA [NM_001166521] | 1,37 | ↑ | 6,67E-04 | 1,41 | ↑ | 4,40E-02 |
| 41 | *rps12* | Bos taurus ribosomal protein S12 (RPS12), mRNA [NM_001014387] | 1,31 | ↑ | 4,57E-02 | 1,32 | ↑ | 3,34E-02 |
| 42 | *ucp3* | Bos taurus uncoupling protein 3 (mitochondrial, proton carrier) (UCP3), mRNA [NM_174210] | 3,24 | ↓ | 4,65E-04 | 2,15 | ↓ | 3,94E-02 |
| 43 | *mstn* | Bos taurus myostatin (MSTN), mRNA [NM_001001525] | 2,89 | ↓ | 2,90E-02 | 2,77 | ↓ | 1,39E-02 |
| 44 | *zmynd8* | Bos taurus zinc finger, MYND-type containing 8 (ZMYND8), mRNA [NM_001191171] | 2,73 | ↓ | 1,09E-02 | 2,29 | ↓ | 4,87E-02 |
| 45 | *csgalnact2* | Bos taurus chondroitin sulfate N-acetylgalactosaminyltransferase 2 (CSGALNACT2), mRNA [NM_001205653] | 2,17 | ↓ | 9,16E-03 | 2,73 | ↓ | 7,02E-03 |
| 46 | *lnpep* | leucyl/cystinyl aminopeptidase [Source:HGNC Symbol;Acc:6656] [ENSBTAT00000026511] | 2,10 | ↓ | 5,12E-03 | 1,86 | ↓ | 1,35E-02 |
| 47 | *tgm6* | transglutaminase 6 [Source:HGNC Symbol;Acc:16255] [ENSBTAT00000002338] | 2,02 | ↓ | 4,82E-02 | 1,98 | ↓ | 3,09E-02 |
| 48 | *atl2* | Bos taurus cDNA clone IMAGE:8270017. [BC120243] | 1,94 | ↓ | 2,89E-02 | 1,38 | ↓ | 1,44E-02 |
| 49 | *sh3bp5* | Bos taurus SH3-domain binding protein 5 (BTK-associated) (SH3BP5), mRNA [NM_001206288] | 1,92 | ↓ | 1,61E-03 | 1,57 | ↓ | 9,12E-03 |
| 50 | *prep* | Bos taurus prolyl endopeptidase, mRNA (cDNA clone MGC:139175 IMAGE:8089735), complete cds. [BC140507] | 1,86 | ↓ | 2,09E-02 | 1,53 | ↓ | 1,63E-02 |
| 51 | *loxl2* | Bos taurus lysyl oxidase-like 2 (LOXL2), mRNA [NM_001099053] | 1,86 | ↓ | 1,96E-02 | 1,53 | ↓ | 9,70E-03 |
| 52 | *ptpn4* | Bos taurus protein tyrosine phosphatase, non-receptor type 4 (megakaryocyte) (PTPN4), mRNA [NM_001192946] | 1,82 | ↓ | 1,93E-02 | 1,85 | ↓ | 3,02E-02 |
| 53 | *ttll1* | Bos taurus tubulin tyrosine ligase-like family, member 1 (TTLL1), mRNA [NM_001076171] | 1,81 | ↓ | 4,25E-02 | 1,50 | ↓ | 3,24E-02 |
| 54 | *st6galnac4* | Bos taurus ST6 (alpha-N-acetyl-neuraminyl-2,3-beta-galactosyl-1,3)-N-acetylgalactosaminide alpha-2,6-sialyltransferase 4 (ST6GALNAC4), mRNA [NM_205791] | 1,81 | ↓ | 2,51E-02 | 1,87 | ↓ | 1,55E-02 |
| 55 | *slc25a39* | Bos taurus solute carrier family 25, member 39 (SLC25A39), mRNA [NM_001075415] | 1,79 | ↓ | 8,35E-03 | 1,47 | ↓ | 3,56E-02 |
| 56 | *camk2d* | Bos taurus calcium/calmodulin-dependent protein kinase II delta (CAMK2D), mRNA [NM_001046333] | 1,73 | ↓ | 3,03E-02 | 1,57 | ↓ | 1,83E-02 |
| 57 | *brms1l* | Bos taurus breast cancer metastasis-suppressor 1-like (BRMS1L), mRNA [NM_001083428] | 1,71 | ↓ | 4,33E-02 | 1,65 | ↓ | 1,95E-02 |
| 58 | *stradb* | Bos taurus STE20-related kinase adaptor beta (STRADB), mRNA [NM_001192081] | 1,69 | ↓ | 3,53E-03 | 1,34 | ↓ | 3,39E-03 |
| 59 | *tbk1* | Bos taurus TANK-binding kinase 1 (TBK1), mRNA [NM_001192755] | 1,68 | ↓ | 8,57E-03 | 1,50 | ↓ | 4,92E-02 |
| 60 | *ppic* | Bos taurus peptidylprolyl isomerase C (cyclophilin C) (PPIC), mRNA [NM_001076910] | 1,66 | ↓ | 2,06E-02 | 1,70 | ↓ | 1,17E-02 |
| 61 | *lum* | Bos taurus lumican (LUM), mRNA [NM_173934] | 1,65 | ↓ | 3,45E-02 | 1,78 | ↓ | 4,21E-02 |
| 62 | *tlk1* | tousled-like kinase 1 [Source:HGNC Symbol;Acc:11841] [ENSBTAT00000009090] | 1,63 | ↓ | 8,06E-03 | 1,85 | ↓ | 1,42E-02 |
| 63 | *det1* | Bos taurus de-etiolated homolog 1 (Arabidopsis) (DET1), mRNA [NM_001099117] | 1,60 | ↓ | 5,04E-04 | 1,52 | ↓ | 2,23E-03 |
| 64 | *ppp2r5e* | Bos taurus protein phosphatase 2, regulatory subunit B', epsilon isoform (PPP2R5E), mRNA [NM_001083468] | 1,60 | ↓ | 1,75E-04 | 1,68 | ↓ | 1,89E-02 |
| 65 | *edem3* | Bos taurus ER degradation enhancer, mannosidase alpha-like 3 (EDEM3), mRNA [NM_001205853] | 1,58 | ↓ | 1,06E-02 | 1,56 | ↓ | 2,42E-02 |
| 66 | *dyrk1a* | Bos taurus dual-specificity tyrosine-(Y)-phosphorylation regulated kinase 1A (DYRK1A), mRNA [NM_001206014] | 1,56 | ↓ | 1,12E-03 | 1,45 | ↓ | 3,75E-02 |
| 67 | *ppm1a* | Bos taurus protein phosphatase, Mg2+/Mn2+ dependent, 1A (PPM1A), mRNA [NM_174429] | 1,54 | ↓ | 2,15E-02 | 1,52 | ↓ | 1,41E-02 |
| 68 | *cct6a* | Bos taurus chaperonin containing TCP1, subunit 6A (zeta 1) (CCT6A), mRNA [NM_001034542] | 1,54 | ↓ | 9,04E-03 | 1,54 | ↓ | 9,26E-03 |
| 69 | *cdc34* | Bos taurus cell division cycle 34 homolog (S. cerevisiae) (CDC34), mRNA [NM_001206236] | 1,53 | ↓ | 3,07E-02 | 1,69 | ↓ | 1,21E-02 |
| 70 | *tgfb2* | Bos taurus transforming growth factor, beta 2 (TGFB2), mRNA [NM_001113252] | 1,52 | ↓ | 9,07E-03 | 1,49 | ↓ | 3,70E-02 |
| 71 | *gsk3b* | Bos taurus glycogen synthase kinase 3 beta (GSK3B), mRNA [NM_001101310] | 1,52 | ↓ | 6,44E-03 | 1,61 | ↓ | 1,24E-02 |
| 72 | *mbtps2* | Bos taurus membrane-bound transcription factor peptidase, site 2 (MBTPS2), mRNA [NM_001075981] | 1,52 | ↓ | 4,40E-03 | 1,32 | ↓ | 2,61E-02 |
| 73 | *slc25a4* | Bos taurus solute carrier family 25 (mitochondrial carrier; adenine nucleotide translocator), member 4 (SLC25A4), mRNA [NM_174658] | 1,51 | ↓ | 1,18E-02 | 1,36 | ↓ | 2,35E-03 |
| 74 | *hspa8* | Bos taurus heat shock 70kDa protein 8 (HSPA8), mRNA [NM_174345] | 1,49 | ↓ | 2,42E-02 | 1,96 | ↓ | 5,24E-04 |
| 75 | *galnt1* | Bos taurus UDP-N-acetyl-alpha-D-galactosamine:polypeptide N-acetylgalactosaminyltransferase 1 (GalNAc-T1) (GALNT1), mRNA [NM_177519] | 1,48 | ↓ | 3,15E-02 | 1,46 | ↓ | 2,07E-02 |
| 76 | *ttll7* | PREDICTED: Bos taurus tubulin tyrosine ligase-like family, member 7 (TTLL7), transcript variant X1, mRNA [XM_005204387] | 1,45 | ↓ | 1,62E-02 | 2,16 | ↓ | 5,19E-03 |
| 77 | *bmpr2* | PREDICTED: Bos taurus bone morphogenetic protein receptor, type II (serine/threonine kinase) (BMPR2), transcript variant 1, mRNA [XM_617592.6] | 1,42 | ↓ | 5,92E-03 | 1,68 | ↓ | 3,67E-02 |
| 78 | *cdk2ap1* | Bos taurus cyclin-dependent kinase 2 associated protein 1 (CDK2AP1), mRNA [NM_001076369] | 1,42 | ↓ | 3,61E-02 | 1,60 | ↓ | 4,24E-02 |
| 79 | *ptp4a1* | Bos taurus protein tyrosine phosphatase type IVA, member 1 (PTP4A1), mRNA [NM_001206124] | 1,41 | ↓ | 4,88E-02 | 1,36 | ↓ | 2,14E-03 |
| 80 | *slc25a26* | Bos taurus solute carrier family 25 (S-adenosylmethionine carrier), member 26 (SLC25A26), mRNA [NM_001103297] | 1,40 | ↓ | 9,28E-03 | 1,43 | ↓ | 2,52E-02 |
| 81 | *cast* | Bos taurus calpastatin (CAST), transcript variant 2, mRNA [NM_174003] | 1,40 | ↓ | 1,19E-02 | 1,44 | ↓ | 1,29E-02 |
| 82 | *large* | Bos taurus like-glycosyltransferase (LARGE), mRNA [NM_001205588] | 1,39 | ↓ | 3,32E-02 | 1,53 | ↓ | 1,75E-02 |
| 83 | *tmem33* | Bos taurus transmembrane protein 33 (TMEM33), transcript variant 2, mRNA [NM_001282453] | 1,39 | ↓ | 2,30E-03 | 1,79 | ↓ | 2,11E-02 |
| 84 | *hbs1l* | Bos taurus HBS1-like (S. cerevisiae) (HBS1L), mRNA [NM_001046498] | 1,39 | ↓ | 2,72E-02 | 1,38 | ↓ | 1,20E-02 |
| 85 | *mapkapk5* | Bos taurus mitogen-activated protein kinase-activated protein kinase 5 (MAPKAPK5), mRNA [NM_001206858] | 1,38 | ↓ | 1,79E-02 | 1,42 | ↓ | 1,51E-02 |
| 86 | *ube2e3* | Bos taurus ubiquitin-conjugating enzyme E2E 3 (UBE2E3), mRNA [NM_001079783] | 1,38 | ↓ | 8,90E-03 | 1,35 | ↓ | 2,17E-02 |
| 87 | *npepps* | Bos taurus aminopeptidase puromycin sensitive (NPEPPS), mRNA [NM_001193159] | 1,38 | ↓ | 4,60E-03 | 1,57 | ↓ | 1,46E-02 |
| 88 | *cdk16* | Bos taurus cyclin-dependent kinase 16 (CDK16), mRNA [NM_001101226] | 1,38 | ↓ | 2,42E-02 | 1,55 | ↓ | 4,60E-02 |
| 89 | *eif4e2* | Bos taurus eukaryotic translation initiation factor 4E family member 2 (EIF4E2), transcript variant 2, mRNA [NM_001206416] | 1,37 | ↓ | 1,32E-02 | 1,38 | ↓ | 6,39E-03 |
| 90 | *cct7* | Bos taurus chaperonin containing TCP1, subunit 7 (eta) (CCT7), mRNA [NM_001046171] | 1,37 | ↓ | 4,98E-04 | 1,31 | ↓ | 1,47E-02 |
| 91 | *prkaa1* | Bos taurus protein kinase, AMP-activated, alpha 1 catalytic subunit (PRKAA1), mRNA [NM_001109802] | 1,36 | ↓ | 2,63E-02 | 1,62 | ↓ | 4,45E-02 |
| 92 | *irak2* | Bos taurus interleukin-1 receptor-associated kinase 2 (IRAK2), mRNA [NM_001075696] | 1,35 | ↓ | 3,94E-02 | 1,82 | ↓ | 4,81E-02 |
| 93 | *abhd11* | Bos taurus abhydrolase domain containing 11 (ABHD11), mRNA [NM_001034372] | 1,35 | ↓ | 3,06E-02 | 1,78 | ↓ | 2,76E-02 |
| 94 | *pias2* | protein inhibitor of activated STAT, 2 [Source:HGNC Symbol;Acc:17311] [ENSBTAT00000010308] | 1,33 | ↓ | 2,28E-02 | 1,32 | ↓ | 1,02E-02 |
| 95 | *ubxn2a* | Bos taurus UBX domain protein 2A (UBXN2A), mRNA [NM_001192967] | 1,33 | ↓ | 6,24E-03 | 2,53 | ↓ | 1,03E-02 |
| 96 | *bmpr1a* | Bos taurus bone morphogenetic protein receptor, type IA (BMPR1A), mRNA [NM_001076800] | 1,33 | ↓ | 1,05E-02 | 1,58 | ↓ | 2,38E-02 |
| 97 | *rps6kc1* | Bos taurus ribosomal protein S6 kinase, 52kDa, polypeptide 1 (RPS6KC1), mRNA [NM_001193022] | 1,31 | ↓ | 4,28E-02 | 1,46 | ↓ | 1,20E-02 |
| 98 | *grk1* | Bos taurus G protein-coupled receptor kinase 1 (GRK1), mRNA [NM_174173] | 1,30 | ↓ | 4,93E-02 | 1,34 | ↓ | 5,32E-05 |
| 99 | *eef1e1* | Bos taurus eukaryotic translation elongation factor 1 epsilon 1 (EEF1E1), mRNA [NM_001040599] | 1,30 | ↓ | 5,56E-03 | 1,47 | ↓ | 1,62E-02 |

**Supplementary Table 3** Classification presenting involvement of the differentially expressed genes in protein metabolism processes (*Pathway Studio)*

| **Biological Processes** | **Count** | **Gene symbol** |
| --- | --- | --- |
| protein synthesis | 36 | *apbb1, apex1, bmpr1a, ccng1, cfb, csgalnact2, ctsd, ddit4, dlc1, dyrk1a, eif4e2, eif6, fbp1, fst, gsk3b, hbs1l, hif1a, hspa8, klf4, map3k5, mstn, myc, prelp, prep, prkaa1, rps12, sepn1, sirt1, slc25a4, smad2, sox17, tgfb2, tgfbi, trim63, ucp3, vrk1* |
| proteolysis | 33 | *adamts1, apbb1, apex1, bcl2l1, c3, cast, cdc34, cdh13, cfb, ctsd, det1, dlc1, fermt2, fst, galnt1, gsk3b, hif1a, hspa8, id1, il1rl1, irak2, lum, masp1, mbtps2, mstn, myc, pard6a, prep, rhbdd1, sirt1, smad2, tgfb2, trim63* |

**Supplementary Table 4** List of differentially expressed genes associated with muscle organ development (*Panther;* FC≥1.3, FC - Fold change; p≤0.05, n=4 for each breed; R - Regulation, direction of expression change was marked with arrow up (increase of expression) and arrow down (expression decrease)

| **No.** | **Gene symbol** | **Description** | **LIM vs. HF** | | | **HER vs. HF** | | |
| --- | --- | --- | --- | --- | --- | --- | --- | --- |
|  |  |  | **FC** | **R** | **p-value** | **FC** | **R** | **p-value** |
| 1 | *adamts1* | Bos taurus ADAM metallopeptidase with thrombospondin type 1 motif, 1 (ADAMTS1), mRNA [NM_001101080] | 2,81 | ↑ | 1,16E-02 | 1,32 | ↑ | 7,44E-04 |
| 2 | *csrp2* | Bos taurus cysteine and glycine-rich protein 2 (CSRP2), mRNA [NM_001038183] | 1,73 | ↑ | 3,13E-02 | 2,14 | ↑ | 4,13E-02 |
| 3 | *myo1d* | Bos taurus myosin ID (MYO1D), mRNA [NM_001075838] | 1,70 | ↑ | 1,09E-02 | 1,71 | ↑ | 4,24E-02 |
| 4 | *fhl2* | Bos taurus four and a half LIM domains 2 (FHL2), mRNA [NM_001046046] | 1,62 | ↑ | 6,57E-03 | 1,77 | ↑ | 7,56E-03 |
| 5 | *fhl3* | Bos taurus four and a half LIM domains 3 (FHL3), mRNA [NM_001034223] | 1,41 | ↑ | 2,35E-02 | 1,78 | ↑ | 1,29E-03 |
| 6 | *csrp1* | Bos taurus cysteine and glycine-rich protein 1 (CSRP1), mRNA [NM_001083771] | 1,36 | ↑ | 2,43E-02 | 1,56 | ↑ | 6,48E-04 |
| 7 | *cdh13* | Bos taurus cadherin 13, H-cadherin (heart) (CDH13), mRNA [NM_001035277] | 1,79 | ↓ | 5,65E-04 | 1,57 | ↓ | 4,88E-02 |
| 8 | *fblim1* | Bos taurus filamin binding LIM protein 1 (FBLIM1), mRNA [NM_001076955] | 1,79 | ↓ | 8,83E-03 | 1,40 | ↓ | 3,70E-02 |

**Supplementary Table 5** Classification presenting involvement of the differentially expressed genes in biological processes associated with muscle organ development (*Pathway Studio)*

| **Biological Processes** | **Count** | **Gene symbol** | **p-value** |
| --- | --- | --- | --- |
| transforming growth factor beta receptor signaling pathway | 14 | *ptprk; smad2; ltbp2; tfdp2; col3a1; tgfbr3; pard6a; id1; bmpr1a; mstn; ppm1a; myc; tgfb2; fermt2* | 2,82E-07 |
| positive regulation of smooth muscle contraction | 4 | *c4a; ptgs1; npnt; sphk1* | 4,25E-04 |
| TGFBR | 3 | *bmpr1a; bmpr2; tgfbr3* | 1,79E-03 |
| regulation of transforming growth factor beta2 production | 2 | *hif1a; tgfb2* | 2,18E-03 |
| negative regulation of cell growth | 9 | *btg1; sirt1; bmpr2; rrad; fbp1; tgfb2; ip6k2; sox17; apbb1* | 2,29E-03 |
| cell proliferation | 17 | *gsk3b; pdk1; ascc3; six2; osmr; tgfbi; bcl2l1; bak1; myc; tgfb2; cfb; uhrf2; il2ra; tacc2; gab1; ddit4; bin1* | 3,47E-03 |
| regulation of cell proliferation | 10 | *tcf7; tes; sirt1; hif1a; bmpr2; sparc; ptgs1; nfkbia; tgfb2; klf4* | 4,47E-03 |
| cardiac muscle cell proliferation | 3 | *tgfbr3; foxs1; tgfb2* | 4,72E-03 |
| skeletal muscle atrophy | 2 | *mstn; trim63* | 5,32E-03 |
| negative regulation of transforming growth factor beta receptor signaling pathway | 5 | *smad2; sirt1; tgfbr3; aspn; ppm1a* | 1,28E-02 |
| negative regulation of muscle hypertrophy | 1 | *mstn* | 1,93E-02 |
| negative regulation of skeletal muscle tissue growth | 1 | *mstn* | 1,93E-02 |
| negative regulation of cell proliferation | 16 | *podn; smad2; tes; btg1; eef1e1; cdh13; bak1; tgfb2; adamts1; pawr; ptprk; aldh1a2; dlc1; il1rl1; ptges; klf4* | 2,13E-02 |
| positive regulation of smooth muscle cell migration | 3 | *camk2d; f3; mmp1* | 2,16E-02 |
| positive regulation of cardiac muscle hypertrophy | 2 | *camk2d; adk* | 2,17E-02 |
| Actomyosin based movement | 4 | *actg1; myo1d; dysf; cdk2ap1* | 2,57E-02 |
| cell growth | 4 | *tgfbr3; ccng1; tgfb2; slc3a2* | 2,88E-02 |
| response to muscle activity | 2 | *hif1a; mstn* | 2,91E-02 |
| negative regulation of smooth muscle cell migration | 2 | *trib1; apex1* | 3,32E-02 |
| positive regulation of skeletal muscle cell proliferation | 1 | *sepn1* | 3,83E-02 |
| negative regulation of muscle hyperplasia | 1 | *klf4* | 3,83E-02 |
| myoblast differentiation | 2 | *cast; csrp2* | 4,66E-02 |

**Supplementary Table 6** List of differentially expressed genes associated with muscle organ development (*Pathway Studio;* FC≥1.3, FC - Fold change; p≤0.05, n=4 for each breed; R - Regulation, direction of expression change was marked with arrow up (increase of expression) and arrow down (expression decrease)

| **No.** | **Gene symbol** | **Description** | **LIM vs. HF** | | | **HER vs. HF** | | |
| --- | --- | --- | --- | --- | --- | --- | --- | --- |
|  |  |  | **FC** | **R** | **p-value** | **FC** | **R** | **p-value** |
| 1 | *fbp1* | Bos taurus fructose-1,6-bisphosphatase 1 (FBP1), mRNA [NM_001034447] | 12,24 | ↑ | 1,55E-02 | 8,71 | ↑ | 2,61E-02 |
| 2 | *myc* | Bos taurus v-myc myelocytomatosis viral oncogene homolog (avian) (MYC), mRNA [NM_001046074] | 11,48 | ↑ | 1,77E-03 | 2,84 | ↑ | 4,83E-02 |
| 3 | *cfb* | Bos taurus complement factor B (CFB), mRNA [NM_001040526] | 6,37 | ↑ | 9,10E-03 | 4,92 | ↑ | 1,03E-02 |
| 4 | *klf4* | Bos taurus Kruppel-like factor 4 (gut) (KLF4), mRNA [NM_001105385] | 5,63 | ↑ | 2,63E-03 | 3,34 | ↑ | 1,89E-02 |
| 5 | *id1* | Bos taurus inhibitor of DNA binding 1, dominant negative helix-loop-helix protein (ID1), mRNA [NM_001097568] | 4,36 | ↑ | 1,45E-03 | 2,10 | ↑ | 4,45E-02 |
| 6 | *rrad* | Bos taurus Ras-related associated with diabetes (RRAD), mRNA [NM_001045913] | 4,25 | ↑ | 8,92E-03 | 4,79 | ↑ | 9,25E-03 |
| 7 | *ltbp2* | Bos taurus latent transforming growth factor beta binding protein 2 (LTBP2), mRNA [NM_174385] | 4,14 | ↑ | 1,14E-02 | 2,37 | ↑ | 1,72E-02 |
| 8 | *trib1* | Bos taurus tribbles homolog 1 (Drosophila) (TRIB1), mRNA [NM_001101105] | 3,94 | ↑ | 2,36E-03 | 2,31 | ↑ | 1,70E-02 |
| 9 | *ddit4* | Bos taurus DNA-damage-inducible transcript 4 (DDIT4), mRNA [NM_001075922] | 3,72 | ↑ | 6,76E-03 | 2,44 | ↑ | 3,93E-02 |
| 10 | *tes* | Bos taurus testis derived transcript (3 LIM domains) (TES), mRNA [NM_001046390] | 3,58 | ↑ | 1,01E-03 | 2,24 | ↑ | 1,12E-02 |
| 11 | *adamts1* | Bos taurus ADAM metallopeptidase with thrombospondin type 1 motif, 1 (ADAMTS1), mRNA [NM_001101080] | 2,81 | ↑ | 1,16E-02 | 1,32 | ↑ | 7,44E-04 |
| 12 | *ptges* | Bos taurus prostaglandin E synthase (PTGES), mRNA [NM_174443] | 2,76 | ↑ | 1,54E-03 | 2,67 | ↑ | 1,82E-02 |
| 13 | *ptprk* | Bos taurus protein tyrosine phosphatase, receptor type, K (PTPRK), mRNA [NM_001191537] | 2,51 | ↑ | 4,68E-02 | 2,84 | ↑ | 1,74E-02 |
| 14 | *btg1* | Bos taurus B-cell translocation gene 1, anti-proliferative (BTG1), mRNA [NM_173999] | 2,32 | ↑ | 3,76E-03 | 2,65 | ↑ | 1,01E-02 |
| 15 | *trim63* | Bos taurus tripartite motif containing 63 (TRIM63), mRNA [NM_001046295] | 2,13 | ↑ | 3,12E-02 | 2,55 | ↑ | 2,81E-02 |
| 16 | *tgfbr3* | PREDICTED: Bos taurus transforming growth factor, beta receptor III (TGFBR3), mRNA [XM_001253071] | 1,96 | ↑ | 6,83E-03 | 1,97 | ↑ | 2,35E-02 |
| 17 | *uhrf2* | Bos taurus ubiquitin-like with PHD and ring finger domains 2 (UHRF2), mRNA [NM_001102270] | 1,93 | ↑ | 2,87E-02 | 1,37 | ↑ | 4,61E-03 |
| 18 | *tgfbi* | Bos taurus transforming growth factor, beta-induced, 68kDa (TGFBI), mRNA [NM_001205402] | 1,90 | ↑ | 1,33E-03 | 1,95 | ↑ | 3,24E-02 |
| 19 | *dlc1* | Bos taurus deleted in liver cancer 1 (DLC1), mRNA [NM_001102493] | 1,75 | ↑ | 2,14E-02 | 1,47 | ↑ | 3,62E-02 |
| 20 | *csrp2* | Bos taurus cysteine and glycine-rich protein 2 (CSRP2), mRNA [NM_001038183] | 1,73 | ↑ | 3,13E-02 | 2,14 | ↑ | 4,13E-02 |
| 21 | *pawr* | PRKC, apoptosis, WT1, regulator [Source:HGNC Symbol;Acc:8614] [ENSBTAT00000061166] | 1,67 | ↑ | 3,62E-02 | 1,50 | ↑ | 4,13E-02 |
| 22 | *pdk1* | Bos taurus pyruvate dehydrogenase kinase, isozyme 1 (PDK1), mRNA [NM_001205957] | 1,64 | ↑ | 1,84E-02 | 1,36 | ↑ | 9,72E-03 |
| 23 | *hif1a* | Bos taurus hypoxia inducible factor 1, alpha subunit (basic helix-loop-helix transcription factor) (HIF1A), mRNA [NM_174339] | 1,60 | ↑ | 4,98E-02 | 1,58 | ↑ | 2,74E-02 |
| 24 | *podn* | PREDICTED: Bos taurus podocan (PODN), transcript variant X1, mRNA [XM_005204538] | 1,55 | ↑ | 1,24E-02 | 1,32 | ↑ | 2,66E-02 |
| 25 | *pard6a* | Bos taurus par-6 partitioning defective 6 homolog alpha (C, elegans) (PARD6A), mRNA [NM_001046274] | 1,50 | ↑ | 1,98E-02 | 1,54 | ↑ | 1,56E-02 |
| 26 | *ip6k2* | Bos taurus inositol hexakisphosphate kinase 2 (IP6K2), mRNA [NM_001034317] | 1,48 | ↑ | 3,29E-02 | 2,22 | ↑ | 3,16E-02 |
| 27 | *osmr* | Bos taurus oncostatin M receptor (OSMR), mRNA [NM_001080272] | 1,47 | ↑ | 4,20E-02 | 1,61 | ↑ | 2,22E-02 |
| 28 | *sirt1* | Bos taurus sirtuin 1 (SIRT1), mRNA [NM_001192980] | 1,46 | ↑ | 4,01E-02 | 1,32 | ↑ | 2,35E-02 |
| 29 | *il2ra* | Bos taurus interleukin 2 receptor, alpha (IL2RA), mRNA [NM_174358] | 1,38 | ↑ | 2,60E-02 | 2,44 | ↑ | 1,94E-03 |
| 30 | *apbb1* | Bos taurus amyloid beta (A4) precursor protein-binding, family B, member 1 (Fe65) (APBB1), mRNA [NM_001075186] | 1,36 | ↑ | 2,55E-02 | 1,57 | ↑ | 2,58E-02 |
| 31 | *bcl2l1* | Bos taurus BCL2-like 1 (BCL2L1), mRNA [NM_001077486] | 1,36 | ↑ | 2,03E-02 | 1,55 | ↑ | 3,92E-02 |
| 32 | *sox17* | Bos taurus SRY (sex determining region Y)-box 17 (SOX17), mRNA [NM_001206251] | 1,35 | ↑ | 2,02E-02 | 1,51 | ↑ | 8,94E-03 |
| 33 | *gab1* | Bos taurus GRB2-associated binding protein 1 (GAB1), mRNA [NM_001101201] | 1,31 | ↑ | 2,30E-02 | 1,86 | ↑ | 6,69E-03 |
| 34 | *sepn1* | Bos taurus selenoprotein N, 1 (SEPN1), mRNA [NM_001114976] | 1,30 | ↑ | 1,09E-02 | 1,50 | ↑ | 4,17E-02 |
| 35 | *col3a1* | Bos taurus collagen, type III, alpha 1 (COL3A1), mRNA [NM_001076831] | 6,11 | ↓ | 2,50E-02 | 3,43 | ↓ | 2,64E-02 |
| 36 | *mstn* | Bos taurus myostatin (MSTN), mRNA [NM_001001525] | 2,89 | ↓ | 2,90E-02 | 2,77 | ↓ | 1,39E-02 |
| 37 | *slc3a2* | Bos taurus solute carrier family 3 (activators of dibasic and neutral amino acid transport), member 2, mRNA (cDNA clone MGC:127541 IMAGE:7950792), complete cds, [BC102420] | 2,34 | ↓ | 1,82E-02 | 2,36 | ↓ | 2,45E-02 |
| 38 | *aldh1a2* | PREDICTED: Bos taurus aldehyde dehydrogenase 1 family, member A2 (ALDH1A2), mRNA [XM_615062] | 2,00 | ↓ | 7,28E-03 | 1,36 | ↓ | 4,31E-02 |
| 39 | *tfdp2* | Bos taurus transcription factor Dp-2 (E2F dimerization partner 2) (TFDP2), mRNA [NM_001075241] | 1,91 | ↓ | 1,17E-02 | 1,41 | ↓ | 1,34E-02 |
| 40 | *six2* | Bos taurus SIX homeobox 2 (SIX2), mRNA [NM_001205678] | 1,81 | ↓ | 4,64E-02 | 1,82 | ↓ | 1,04E-03 |
| 41 | *cdh13* | Bos taurus cadherin 13, H-cadherin (heart) (CDH13), mRNA [NM_001035277] | 1,79 | ↓ | 5,65E-04 | 1,57 | ↓ | 4,88E-02 |
| 42 | *il1rl1* | Bos taurus interleukin 1 receptor-like 1 (IL1RL1), mRNA [NM_001206302] | 1,67 | ↓ | 3,30E-02 | 2,03 | ↓ | 4,63E-02 |
| 43 | *ascc3* | Bos taurus activating signal cointegrator 1 complex subunit 3 (ASCC3), mRNA [NM_001206118] | 1,55 | ↓ | 6,67E-03 | 1,66 | ↓ | 2,15E-03 |
| 44 | *ppm1a* | Bos taurus protein phosphatase, Mg2+/Mn2+ dependent, 1A (PPM1A), mRNA [NM_174429] | 1,54 | ↓ | 2,15E-02 | 1,52 | ↓ | 1,41E-02 |
| 45 | *aspn* | Bos taurus asporin (ASPN), mRNA [NM_001034309] | 1,54 | ↓ | 1,51E-02 | 1,92 | ↓ | 2,23E-02 |
| 46 | *fermt2* | Bos taurus fermitin family member 2 (FERMT2), mRNA [NM_001101264] | 1,53 | ↓ | 1,15E-02 | 1,63 | ↓ | 1,04E-03 |
| 47 | *tgfb2* | Bos taurus transforming growth factor, beta 2 (TGFB2), mRNA [NM_001113252] | 1,52 | ↓ | 9,07E-03 | 1,49 | ↓ | 3,70E-02 |
| 48 | *gsk3b* | Bos taurus glycogen synthase kinase 3 beta (GSK3B), mRNA [NM_001101310] | 1,52 | ↓ | 6,44E-03 | 1,61 | ↓ | 1,24E-02 |
| 49 | *smad2* | Bos taurus SMAD family member 2 (SMAD2), mRNA [NM_001046218] | 1,49 | ↓ | 2,38E-03 | 1,32 | ↓ | 3,32E-02 |
| 50 | *apex1* | Bos taurus APEX nuclease (multifunctional DNA repair enzyme) 1 (APEX1), mRNA [NM_176609] | 1,45 | ↓ | 2,22E-03 | 1,32 | ↓ | 4,23E-02 |
| 51 | *bak1* | Bos taurus BCL2-antagonist/killer 1 (BAK1), mRNA, complete cds, [BT030701] | 1,44 | ↓ | 2,02E-02 | 1,56 | ↓ | 5,01E-03 |
| 52 | *bin1* | Bos taurus cDNA clone IMAGE:8304839, [BC149441] | 1,44 | ↓ | 4,91E-02 | 1,47 | ↓ | 3,50E-02 |
| 53 | *tacc2* | TACC2 protein; Uncharacterized protein [Source:UniProtKB/TrEMBL;Acc:A6QNY2] [ENSBTAT00000061158] | 1,44 | ↓ | 1,23E-02 | 1,36 | ↓ | 8,47E-03 |
| 54 | *ccng1* | Bos taurus cyclin G1 (CCNG1), mRNA [NM_001013364] | 1,43 | ↓ | 8,31E-05 | 1,56 | ↓ | 2,42E-03 |
| 55 | *bmpr2* | PREDICTED: Bos taurus bone morphogenetic protein receptor, type II (serine/threonine kinase) (BMPR2), transcript variant 1, mRNA [XM_617592.6] | 1,42 | ↓ | 5,92E-03 | 1,68 | ↓ | 3,67E-02 |
| 56 | *cast* | Bos taurus calpastatin (CAST), transcript variant 2, mRNA [NM_174003] | 1,40 | ↓ | 1,19E-02 | 1,44 | ↓ | 1,29E-02 |
| 57 | *bmpr1a* | Bos taurus bone morphogenetic protein receptor, type IA (BMPR1A), mRNA [NM_001076800] | 1,33 | ↓ | 1,05E-02 | 1,58 | ↓ | 2,38E-02 |
| 58 | *eef1e1* | Bos taurus eukaryotic translation elongation factor 1 epsilon 1 (EEF1E1), mRNA [NM_001040599] | 1,30 | ↓ | 5,56E-03 | 1,47 | ↓ | 1,62E-02 |
